# Supercritical carbon dioxide extraction of astaxanthin from *Corynebacterium glutamicum*

**DOI:** 10.1186/s40643-025-00882-9

**Published:** 2025-05-26

**Authors:** Jan Seeger, Maximilian Zäh, Volker F. Wendisch, Christoph Brandenbusch, Nadja A. Henke

**Affiliations:** 1 Genetics of Prokaryotes, Faculty of Biology & CeBiTec, Bielefeld University, Bielefeld, Germany; 2 Laboratory of Thermodynamics, TU Dortmund University, Dortmund, Germany; 3 Present address: Institute for Process Engineering in Life Sciences, Karlsruhe Institute of Technology (KIT), Karlsruhe, Germany

**Keywords:** Astaxanthin, Supercritical carbon dioxide, Extraction, *Corynebacterium glutamicum*

## Abstract

Astaxanthin, a red carotenoid with potent antioxidant properties, holds significant value in the feed, cosmetics, and nutraceutical industries. While traditionally sourced from microalgae, *Corynebacterium glutamicum*, a well-established industrial microorganism, has been engineered to serve as an efficient host for astaxanthin production. As astaxanthin integrates into the cellular membrane, effective extraction methods are essential to access this valuable compound. In this study, a sustainable batch extraction process using supercritical carbon dioxide (scCO₂) as a green solvent was developed. The effects of cosolvent concentration (0–9% (*w*/*w*)), temperature (50–75 °C), and pressure (450–650 bar) were investigated with regard to the extraction yield. An optimized extraction was achieved with 9% (*w*/*w*) ethanol as a cosolvent, at 68 °C and 550 bar, allowing the extraction of 67.5 ± 3.7% of the cellular astaxanthin within 0.5 h. Prolonging the extraction time further increased the recovery to 93.3%, which is comparable to processes that have been established for the extraction of astaxanthin from microalgae and yeast. This approach provides a scalable and environmentally friendly solution for industrial astaxanthin recovery.

**Graphical Abstract:**

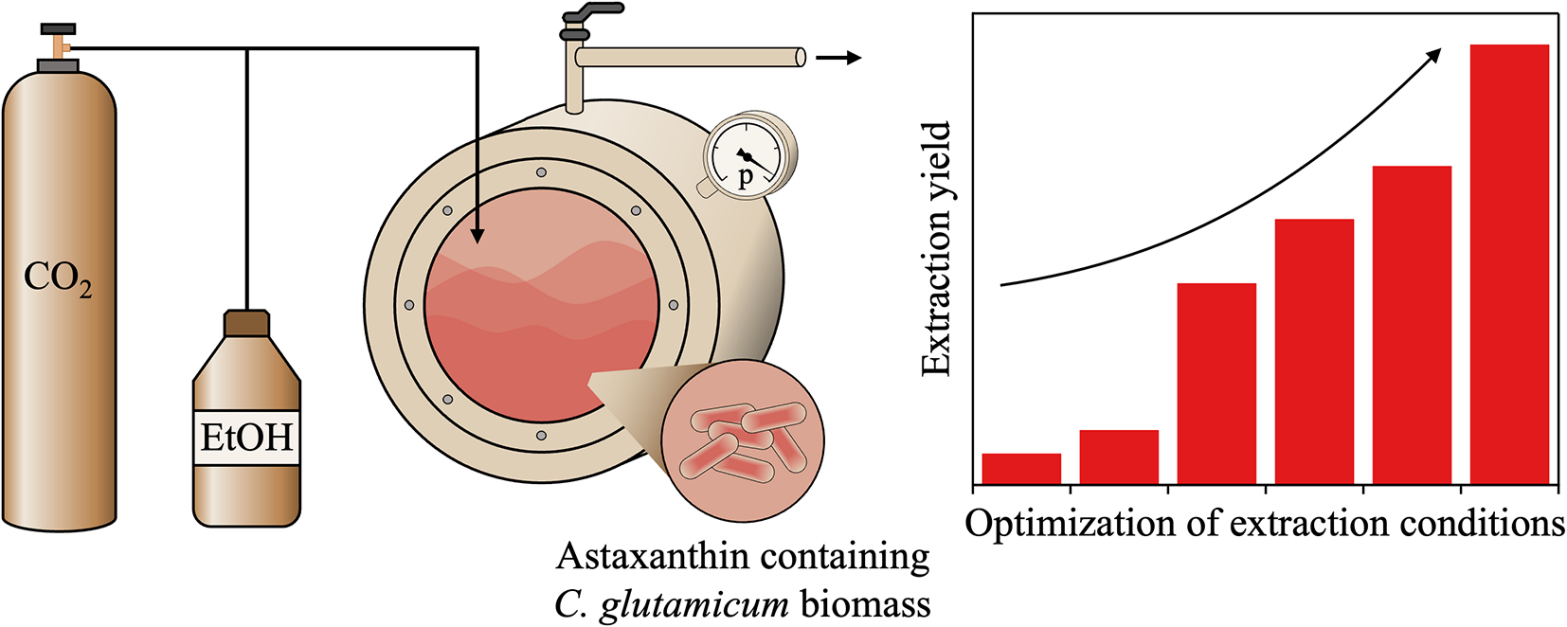

**Supplementary Information:**

The online version contains supplementary material available at 10.1186/s40643-025-00882-9.

## Introduction

Although more than 1,100 carotenoids occur in nature (Yabuzaki 2017), only a few are industrially relevant (Sathasivam and Ki 2018). Among them is the red-colored marine carotenoid astaxanthin with a current market size of USD 2.83 bn and an expected compound annual growth rate of 17.1% until 2030 (Astaxanthin Market 2024). Besides its application as an animal feed additive (Lim et al. 2018), astaxanthin is being more and more used in the nutraceutical and cosmetics industries due to its various health promoting effects (Galasso et al. 2017; Barreiro and Barredo 2018; Stachowiak and Szulc 2021). However, only natural astaxanthin can be used for the latter, as the usage of synthetic astaxanthin is not considered for human consumption (Li et al. 2011). The most common microbial production systems for natural astaxanthin are the microalgae *Haematococcus pluvialis* (Rodríguez-Sifuentes et al. 2020; An et al. 2024), the red yeast *Xanthophyllomyces dendrorhous* (Zhuang and Zhu 2021), and the Gram-negative bacterium *Paracoccus carotinifaciens* (Hayashi et al. 2021). Based on its lipophilic nature, astaxanthin (and its esterified derivatives) are either incorporated into the cellular membrane (Kishimoto et al. 2016) or stored in intracellular lipid droplets (Ota et al. 2018). Due to the relatively rigid cell envelope of microalgae and yeasts, it is generally necessary to permeabilize or disrupt the cells before extraction (Rodríguez-Sifuentes et al. 2020). This can be achieved using physical methods like bead milling (Molino et al. 2018b; Irshad et al. 2019) or high-pressure homogenization (Praveenkumar et al. 2020), chemical methods such as acid treatment (Sarada et al. 2006; Wu et al. 2011), or biological methods like enzymatic lysis (Machado et al. 2016; Harith et al. 2020). Different extraction processes have been developed, comprising (pressurized) organic solvents, e.g., acetone, ethanol, and ethyl acetate (Molino et al. 2018b; Irshad et al. 2019; Praveenkumar et al. 2020), vegetable oils (Kang and Sim 2008), ionic liquids (Desai et al. 2016), and eutectic solvents (Pitacco et al. 2022).

Another solvent-based method for the extraction of astaxanthin is the supercritical fluid extraction (SFE). By reaching the supercritical point, the properties of the gas and the liquid converge, leading to a state characterized by (low) gas-like surface tension, diffusivity and viscosity, and liquid-like density (Knez et al. 2014). Being non-toxic, non-flammable, chemically stable as well as readily affordable, carbon dioxide (CO_2_) is considered as a green solvent (Wu and Han 2019) making it the most common used solvent for SFE in food processing (Picot-Allain et al. 2021). The rather low supercritical point (*T*_*C*_ = 31.1 °C, *p*_*C*_ = 73.8 bar) of CO_2_ (Wu and Han 2019) enables an extraction under mild conditions, thus avoiding thermal or chemical degradation of the extract (Picot-Allain et al. 2021). The main factor affecting the solvent power of supercritical CO_2_ (scCO_2_) is its density, which can be adjusted by pressure and temperature (Knez and Lütge 2023). Increasing the pressure increases the density, favoring the solubility of the solute. Conversely, higher temperatures elevate the solute’s vapor pressure while reducing the solvent’s density. Therefore, optimization of the process conditions is required for an efficient extraction process (Knez et al. 2014; Wang et al. 2021). The polarity of the scCO_2_ can be modified by cosolvents (polar modifier) like polar organic solvents or plant oils (Krichnavaruk et al. 2008; Wang et al. 2021). After the extraction, simple pressure reduction enables the residue-free removal of the CO_2_ from the extract (Kang et al. 2024). By employing multiple separators and gradually reducing pressure (and thus solvent power of scCO_2_), different fractions of the extract can be collected (Knez and Lütge 2023). Recovery of the gas after expansion allows its recycling without the need of solvent purification (Knez et al. 2014; Kang et al. 2024). Apart from being used for the extraction of essential oils, phenolic compounds, and alkaloids from natural sources (Wang et al. 2021), scCO_2_ extraction has been applied and optimized for the extraction of astaxanthin from *H. pluvialis* in numerous studies (Valderrama et al. 2003; Machmudah et al. 2006; Nobre et al. 2006; Krichnavaruk et al. 2008; Pan et al. 2012; Reyes et al. 2014; Di Sanzo et al. 2018; Molino et al. 2018a; Álvarez et al. 2020). Besides extraction from microalgae, scCO_2_ extraction of astaxanthin has been also achieved from yeast (Lim et al. 2002; Harith et al. 2020), shrimp (Ahmadkelayeh et al. 2022), Gram-negative bacteria (Chougle et al. 2016), and oilseed (Xie et al. 2019).

Renowned for the large-scale production of amino acids (Wendisch 2020), the Gram-positive soil bacterium *Corynebacterium glutamicum* has been proven to be a promising alternative to the aforementioned organisms for astaxanthin production. Harnessing its native carotenoid metabolic pathway, astaxanthin biosynthesis was enabled and improved in several studies, resulting in a titer of 103 mg/L astaxanthin in fed-batch fermentation (Henke et al. 2016; Henke and Wendisch 2019; Göttl et al. 2024). Recently, an extraction process based on ethanol was developed, resulting in a 94% recovery of astaxanthin (Seeger et al. 2023). An in vitro assay revealed a superior antioxidant activity of the obtained natural extract (astaxanthin oleoresin) compared to synthetic astaxanthin and a similar activity to microalgae-derived astaxanthin (Seeger et al. 2023). This study establishes a scCO₂-based extraction process for astaxanthin from *C. glutamicum*, aiming to provide an environmentally friendly and sustainable alternative that minimizes reliance on toxic organic solvents, thereby supporting advancements in the bio-economy.

## Materials and methods

### Chemicals

All chemicals were purchased from Carl Roth (Karlsruhe, Germany) or Sigma-Aldrich (St. Louis, MO, US). Organic solvents for extraction and analysis were HPLC grade. Carbon dioxide (99.9995% (*v*/*v*)) was purchased from Messer (Bad Soden am Taunus, Germany).

### Cultivation and harvesting of *Corynebacterium glutamicum*

The astaxanthin producing strain *Corynebacterium glutamicum* ASTA* was cultivated as described in Henke and Wendisch (2019). After 48 h of cultivation, the cells were harvested by centrifugation at 10,000 x g for 20 min and were oven-dried at 50 °C. The dried biomass contained 0.35 mg/g astaxanthin (quantification described in Sect. 2.5).

### Experimental apparatus

A high-pressure variable-volume view cell (HPVVV; *p*_max_ = 700 bar, *T*_max_ = 180 °C; New Ways of Analytics, Lörrach, Germany) was used for extraction. The required pressure was produced by adjusting the volume using a manual hydraulic press M(O) 189 (Maximator, Zorge, Germany). The system was homogenized by a stirrer. The temperature was measured inside the view cell and was regulated by a heating jacket. If applicable, ethanol was added through a 1/8″ port. A 260D syringe pump (Teledyne ISCO, Lincoln, NE, US) (*p*_max_ = 560 bar) was used for dosing compressed CO_2_ into the HPVVV.

### Experimental procedure

#### scCO_2_ extraction without cosolvent

The dried biomass (see Sect. 2.2) was weighted, packed into a paper tea bag and positioned into the HPVVV. After sealing the HPVVV, CO_2_ was loaded reaching a mass fraction of ≥ 0.99 compared to the biomass. Temperature and pressure were adjusted accordingly (see supplementary Table S1 for all tested conditions). If not stated differently, the extraction was terminated after 0.5 h by releasing the scCO_2_ from the HPVVV. The biomass was taken out of the tea bag and subsequently processed for (residual) astaxanthin analysis (see supplementary Figure S1 for schematic experimental procedure).

#### scCO_2_ extraction with cosolvent

Experimental procedure and loading of the cell with biomass was performed as described above. The cosolvent (ethanol) was added after the HPVVV front sapphire had been sealed. The CO_2_ amount was introduced as in the previous case and temperature and pressure were adjusted accordingly (see supplementary Table S1 for all tested conditions).

### Quantification of astaxanthin content by HPLC

To determine the astaxanthin content of the biomass, a defined amount was extracted with 1 mL of a 7:3 (*v*/*v*) mixture of methanol:acetone at 1000 rpm and 60 °C for 0.5 h using the ThermoMixer C (Eppendorf, Hamburg, Germany). After centrifugation for 10 min at 20,000 x g, the supernatant was analyzed via HPLC. The Agilent 1200 series (Agilent Technologies, Santa Clara, CA, US) equipped with a reversed-phase precolumn (LiChrospher 100 RP18 EC-5, 40 × 4 mm) (CS-Chromatographie, Langerwehe, Germany), a reversed-phase main column (LiChrospher 100 RP18 EC-5, 125 × 4 mm) (CS-Chromatographie, Langerwehe, Germany), and a diode array detector (DAD) was used for analysis. Methanol:water (9:1) (A) and methanol (B) were used as mobile phases. The injection volume was 50 µL, and a gradient at a flow rate of 1.5 mL min^–1^ was used as the following: 0 min B: 0%, 10 min B: 100%, and 32.5 min B: 100%. Carotenoids were quantified by recording the absorption at λ = 470 nm. Astaxanthin (Sigma-Aldrich, St. Louis, MO, US), adonirubin (CaroteNature, Münsingen, Switzerland), canthaxanthin (VWR, Darmstadt, Germany), echinenone (Sigma-Aldrich, St. Louis, MO, US), β-carotene (Sigma-Aldrich, St. Louis, MO, US), and lycopene (ExtraSynthese, Genay, France) were used as reference standards for quantification of each carotenoid in the extract. Exemplary HPLC chromatograms are shown in the supplementary Figure S2. The extraction yield of each carotenoid was calculated individually according to Eq. (1) with the total extracted amount calculated by comparing the initial content of the respective carotenoid in the biomass with its content after the scCO_2_ extraction (Eq. 2).

1 $$\eqalign{&\varvec{E}\varvec{x}\varvec{t}\varvec{r}\varvec{a}\varvec{c}\varvec{t}\varvec{i}\varvec{o}\varvec{n}\:\varvec{y}\varvec{i}\varvec{e}\varvec{l}\varvec{d}\:\left[\varvec{\%}\right]=\cr&\left(\frac{\varvec{E}\varvec{x}\varvec{t}\varvec{r}\varvec{a}\varvec{c}\varvec{t}\varvec{e}\varvec{d}\:\varvec{c}\varvec{a}\varvec{r}\varvec{o}\varvec{t}\varvec{e}\varvec{n}\varvec{o}\varvec{i}\varvec{d}\:\left[\varvec{m}\varvec{g}\:{\varvec{g}}^{-1}\right]}{{\varvec{C}\varvec{a}\varvec{r}\varvec{o}\varvec{t}\varvec{e}\varvec{n}\varvec{o}\varvec{i}\varvec{d}}_{\varvec{b}\varvec{e}\varvec{f}\varvec{o}\varvec{r}\varvec{e}\:\varvec{e}\varvec{x}\varvec{t}\varvec{r}\varvec{a}\varvec{c}\varvec{t}\varvec{i}\varvec{o}\varvec{n}}\left[\varvec{m}\varvec{g}\:{\varvec{g}}^{-1}\right]}\:\times\:100\right)\cr}$$

With

2 $$\eqalign{&\varvec{E}\varvec{x}\varvec{t}\varvec{r}\varvec{a}\varvec{c}\varvec{t}\varvec{e}\varvec{d}\:\varvec{c}\varvec{a}\varvec{r}\varvec{o}\varvec{t}\varvec{e}\varvec{n}\varvec{o}\varvec{i}\varvec{d}\cr&=\:{\varvec{C}\varvec{a}\varvec{r}\varvec{o}\varvec{t}\varvec{e}\varvec{n}\varvec{o}\varvec{i}\varvec{d}}_{\varvec{b}\varvec{e}\varvec{f}\varvec{o}\varvec{r}\varvec{e}\:\varvec{e}\varvec{x}\varvec{t}\varvec{r}\varvec{a}\varvec{c}\varvec{t}\varvec{i}\varvec{o}\varvec{n}}\left[\varvec{m}\varvec{g}\:{\varvec{g}}^{-1}\right]-\:\cr&{\varvec{C}\varvec{a}\varvec{r}\varvec{o}\varvec{t}\varvec{e}\varvec{n}\varvec{o}\varvec{i}\varvec{d}}_{\varvec{a}\varvec{f}\varvec{t}\varvec{e}\varvec{r}\:\varvec{e}\varvec{x}\varvec{t}\varvec{r}\varvec{a}\varvec{c}\varvec{t}\varvec{i}\varvec{o}\varvec{n}}\left[\varvec{m}\varvec{g}\:{\varvec{g}}^{-1}\right]\cr}$$

## Results

For the establishment of a scCO_2_-based extraction process of astaxanthin from corynebacterial biomass, three parameters were considered and optimized. (I) the impact of ethanol as a cosolvent, (II) the impact of extraction temperature, and lastly, the impact of (III) extraction time under optimized process conditions.

### Impact of cosolvent addition

**Fig. 1:**
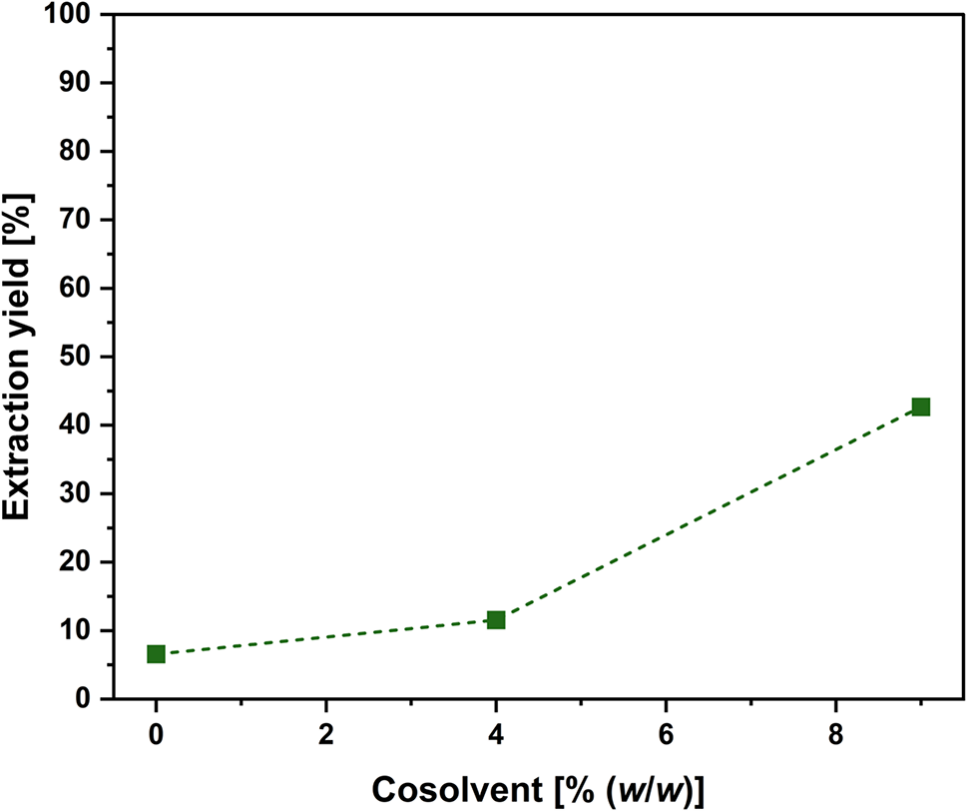
Effect of cosolvent on the astaxanthin extraction yield. Extractions were performed at 550 bar and 55 °C for 0.5 h as single replicates. Ethanol was added as a cosolvent with indicated concentrations

Available literature data on the scCO_2_ extraction of astaxanthin from microalgal biomass revealed the requirement of using a cosolvent for extraction (Machmudah et al. 2006; Nobre et al. 2006; Reyes et al. 2014). Furthermore, extraction temperature and pressure surpassing 50 °C and 500 bar were needed for efficient extraction in several studies (Di Sanzo et al. 2018; Molino et al. 2018a; Álvarez et al. 2020). Based on that, the effect of ethanol as a cosolvent on the extraction of astaxanthin was investigated at 550 bar and 55 °C for 0.5 h. Experiments were conducted as described in the materials and methods section. Without cosolvent, an astaxanthin extraction yield of 6.6% was achieved. The extraction yield increased to 11.5% and 42.7% upon the addition of 4% (*w*/*w*) and 9% (*w*/*w*) ethanol, respectively (Fig. 1). Due to the strong positive effect of the cosolvent, 9% (*w*/*w*) ethanol was used for all following experiments.

### Impact of extraction temperature

**Fig. 2:**
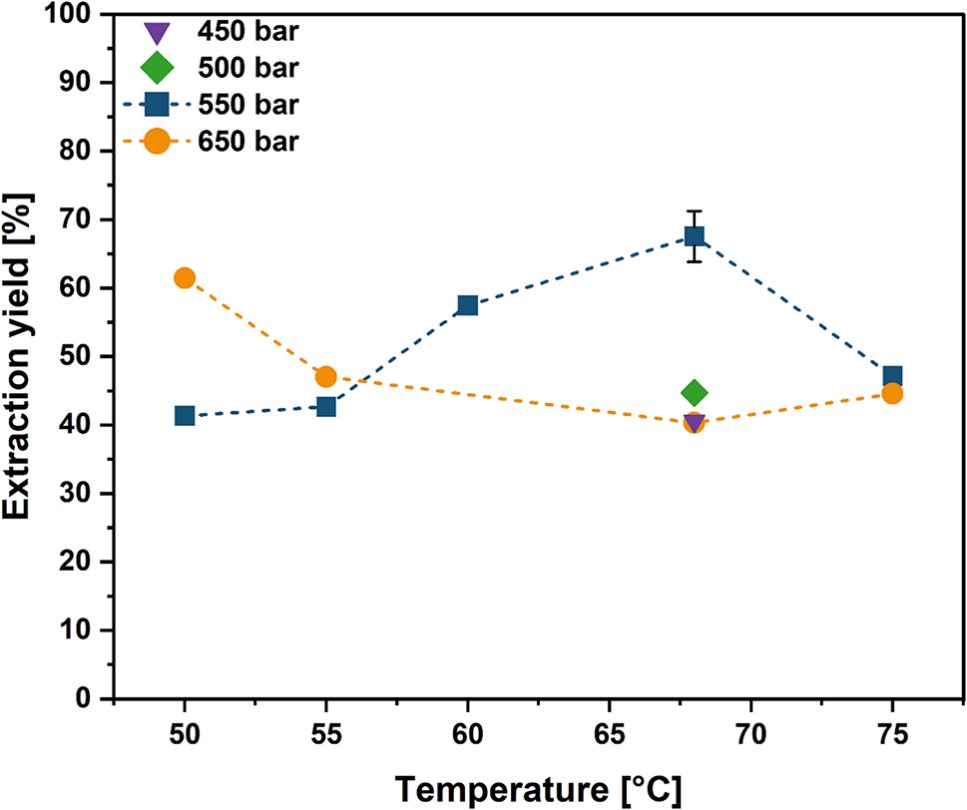
Effect of temperature on astaxanthin extraction yield. Extractions were performed for 0.5 h at 450 bar (purple), 500 bar (green), 550 bar (blue) and 650 bar (orange) at the indicated temperatures. Single replicates were conducted except for the extraction at 68 °C and 550 bar (*n* = 3; mean ± sd)

In the next step, different extraction temperatures were screened at two different pressures (Fig. 2*).* Applying 550 bar, the extraction yield profile showed a clear optimum at 68 °C with 67.5 ± 3.7% of astaxanthin being successfully extracted. In contrast, varying the temperature at 650 bar showed a decreased extraction yield with increasing temperature. For 650 bar, the maximal extraction yield of 61.5% was achieved at 50 °C. Since high pressure did not improve the extraction, 450 bar and 500 bar were tested at 68 °C resulting in a decreased extraction yield.

### Effect of extraction time

**Fig. 3:**
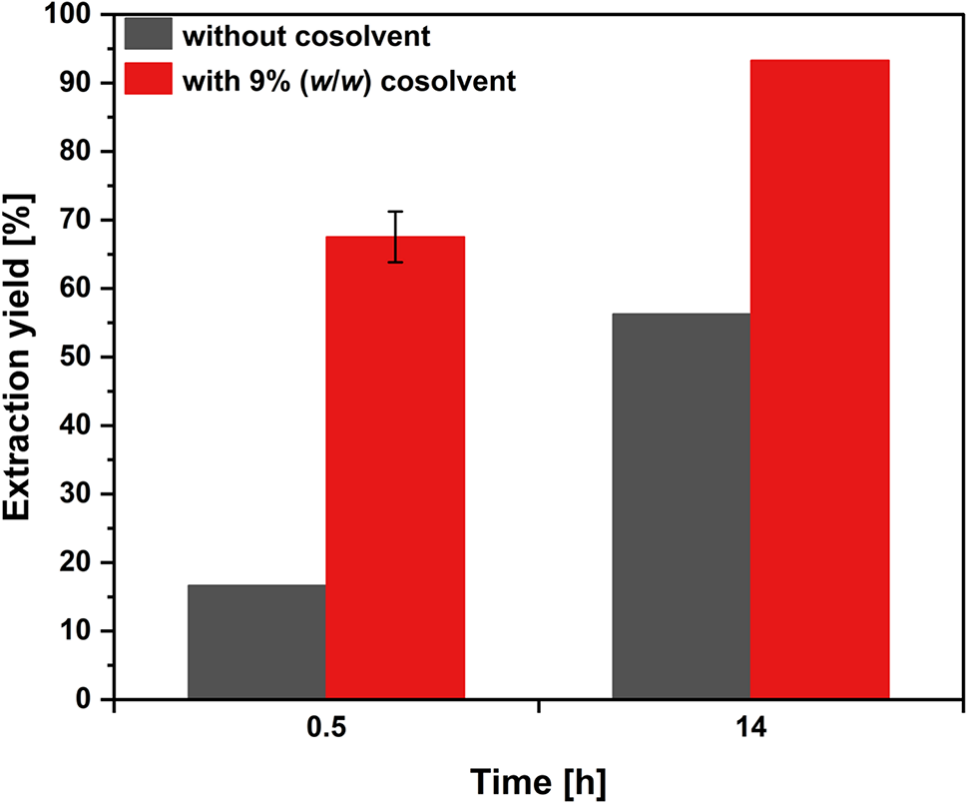
Effect of process time on astaxanthin extraction yield. Extractions were performed at 550 bar and 68 °C, with (red) and without (grey) ethanol as cosolvent for the indicated time. Single replicates were conducted except for the extraction at 0.5 h with cosolvent (*n* = 3; mean ± sd)

As it is well known that extraction time is crucial when considering scCO_2_ extraction from biomass, the extraction time was extended up to 14 h to allow for prolonged penetration of the cellular membranes with scCO_2_. The effect of an extended process time was analyzed for both conditions, with and without the addition of ethanol as cosolvent (Fig. 3). Without the cosolvent, the extraction yield increased from 16.6% after 0.5 h to 56.3% after 14 h of process time. A similar effect was observed for the condition with cosolvent. The extended extraction time increased the extraction yield from 67.5 ± 3.7% to 93.3%.

### Effect of extraction protocol on carotenoid composition

**Fig. 4:**
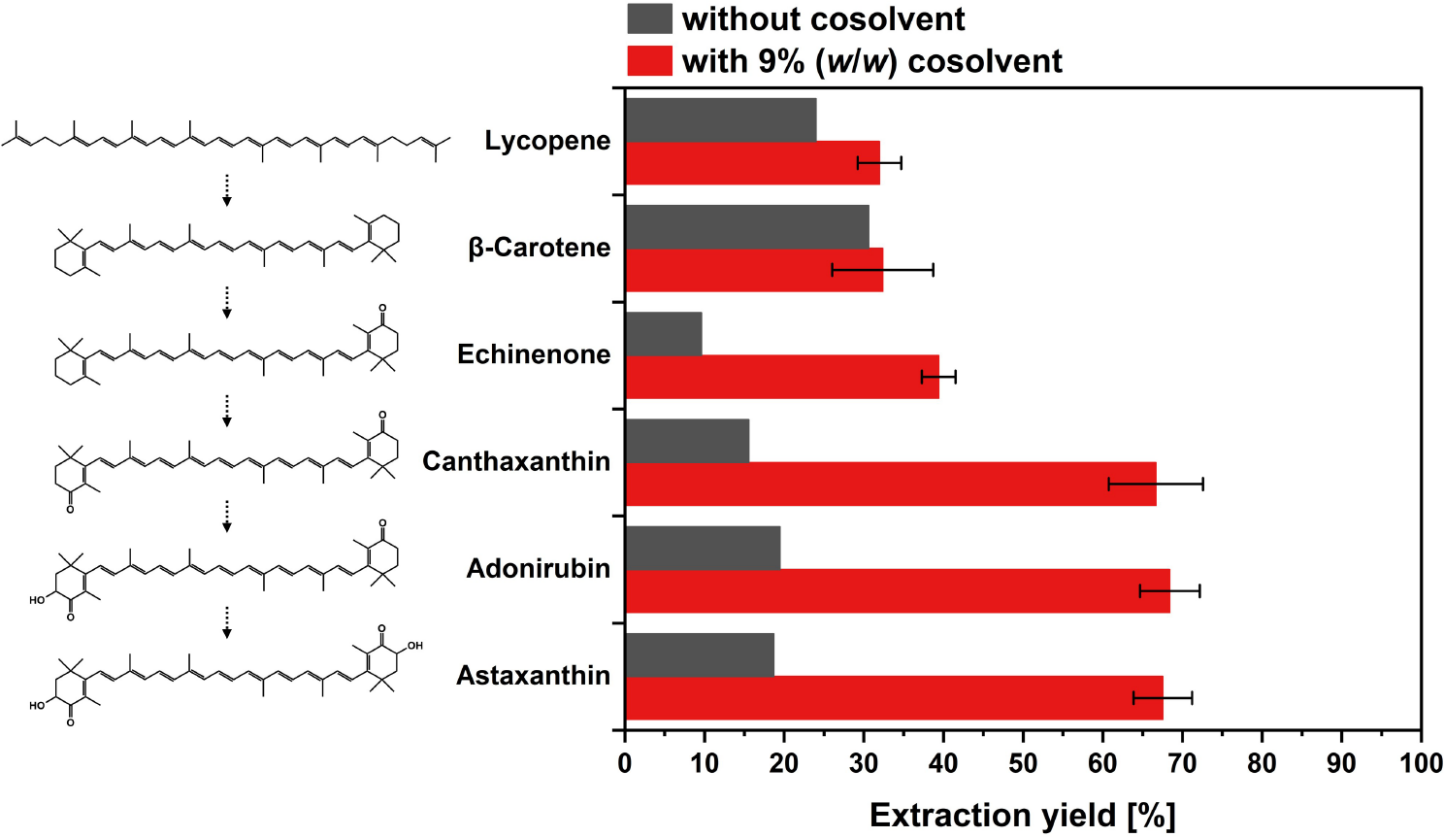
Extracted carotenoids. Extractions were performed at 550 bar and 68 °C for 0.5 h without (grey) and with the addition of 9% (*w*/*w*) ethanol as a cosolvent (red; *n* = 3; mean ± sd). The respective carotenoid structures are shown; the arrows indicate the chronological order of the astaxanthin biosynthesis pathway starting from lycopene

The biomass examined in this study contained astaxanthin as the main product with 40.7% (*w*/*w*) of the total carotenoid content. The remaining 59.3% (*w*/*w*) of the cellular carotenoids were composed of different carotenoid intermediates of the astaxanthin biosynthetic pathway (see Figure S2). It was thus investigated, which other carotenoids, despite astaxanthin, are preferably extracted and thus present in the obtained extract. The extraction yields of the different carotenoids with and without the use of ethanol as cosolvent were assessed (Fig. 4). Without the cosolvent, preferentially lycopene and β-carotene were extracted compared to the oxy-functionalized carotenoids astaxanthin, adonirubin, canthaxanthin and echinenone. This pattern switched upon adding the cosolvent, resulting in a better extractability of the xanthophylls. In both cases, the total extracted amounts of astaxanthin, adonirubin and canthaxanthin were comparable.

## Discussion

Among the different methods available for astaxanthin extraction, scCO_2_ extraction is probably the most studied one as this is the method of choice for large scale extraction of astaxanthin from microalgae (Rodríguez-Sifuentes et al. 2020). Although scCO_2_ extraction has also been investigated for some alternative astaxanthin sources, the available data for the extraction from bacterial sources is limited. However, bacterial processes for astaxanthin production are emerging (Park et al. 2018; Hasunuma et al. 2019; Diao et al. 2020), consequently, requiring appropriate extraction methods for product recovery. In this study, different process parameters of batchwise scCO_2_ extraction were screened to optimize the extraction yield of astaxanthin from corynebacterial biomass.

The addition of 9% (*w*/*w*) ethanol increased the extraction yield from initial 6.6% to 42.7%. This finding is in good agreement with several studies that found the addition of ethanol as a cosolvent to be beneficial for the astaxanthin recovery from microalgae (Valderrama et al. 2003; Machmudah et al. 2006; Nobre et al. 2006; Pan et al. 2012; Reyes et al. 2014). This improvement is caused by two reasons. First, scCO_2_ is a highly apolar solvent (Kang et al. 2024). By addition of ethanol, the solvent mixture becomes more polar, thus favoring the solubility of astaxanthin. The inclusion of a polar cosolvent is particularly relevant for corynebacterial astaxanthin, surpassing its importance in the case of astaxanthin derived from microalgae. Unlike astaxanthin from microalgae, which is esterified with apolar fatty acids, the astaxanthin investigated in this study exists in its free form (Kumar et al. 2022), exhibiting a higher polarity. The relation between solute and solvent polarity becomes apparent in Fig. 4. Without a cosolvent, the carotenes lycopene and β-carotene, consisting of just pure hydrocarbon, showed a better extraction yield than the xanthophylls (astaxanthin, adonirubin, canthaxanthin, and echinenone), which possess varying degrees of oxy-functionalization. These differences in solubility were also observed and discussed by de la Fuente et al. (2006). Upon modifying the polarity with ethanol, the extraction yield of astaxanthin and the xanthophylls increased (Fig. 4). A similar observation was reported by Montero et al. (2005), who also assigned the improved extraction of different xanthophylls to the increased polarity. The second reason for an improvement extraction using a cosolvent might be based on the swelling of the matrix, which in turn increases the contact area with the scCO_2_ (Moore and Taylor 1996; Lim et al. 2002; Nobre et al. 2006). The amount of ethanol was limited to 9% (*w/w*), as high concentrations of the cosolvent were previously shown to reduce/negatively affect the density of the scCO_2_ as well as the selectivity of the extraction (Machmudah et al. 2006).

Next, the extraction temperature was optimized at 550 bar and 650 bar. At lower temperatures, higher pressure was favorable for the extraction yield. This can be explained by the higher density of the scCO_2_ with increasing pressure, thus leading to a higher solvent power (Knez et al. 2014; Wang et al. 2021). The extraction yield at 650 bar decreased with increasing temperature, due to the decreased density at higher temperatures. However, at 550 bar, the extraction yield increased with increasing temperature, reaching a maximum of 67.5 ± 3.7% at 68 °C. The observed optimum is in the same range as the optimum determined by Machmudah et al. (2006) (69.9 °C, 550 bar) and by Molino et al. (2018a) (65 °C, 550 bar). It appears that the extraction yield is more dependent on the temperature, and thus on the vapor pressure of the solute, than on the scCO_2_ density, which is consistent with some observations from the literature (de la Fuente et al. 2006; Álvarez et al. 2020). The effect of temperature becomes also apparent when comparing the extraction yields at different temperatures at 550 bar without a cosolvent. The extraction yield increased from 6.6% at 55 °C (Fig. 1) to 18.7% at 68 °C (Fig. 4). As the extraction yield decreased with decreasing pressure, the optimal balance between fluid density and solute vapor pressure was at 68 °C and 550 bar.

A maximum mass fraction of 4*10^− 7^ (without cosolvent) and 2*10^− 6^ ± 5*10^− 7^ (with cosolvent) has been achieved with the aforementioned optimum. This is up to two magnitudes lower than the values reported by de la Fuente et al. (2006) (2*10^− 6^, 300 bar, 40 °C) and Youn et al. (2007) (7*10^− 4^, 300 bar, 60 °C). Therefore, the limiting factor for the total amount of astaxanthin extracted from biomass is not the solubility, as the equilibrium is still far away from being reached.

Extraction processes can be divided into a solubility- and diffusion-controlled mass transfer period (Knez et al. 2010). As the astaxanthin solubility was not reached even after 14 h (Fig. 3), it is plausible that the extraction is diffusion-controlled/limited. Shortening the diffusion path through, e.g., reduced particle size or by cell disruption, can prospectively reduce the extraction time (Knez et al. 2010). Although cell disruption upon scCO_2_ treatment has been observed for fungi and bacteria (Hossain et al. 2015; Primožič et al. 2019), this effect was already shown for diffusion-controlled extraction processes where cell disruption improved the astaxanthin extraction (Valderrama et al. 2003; Nobre et al. 2006), or smaller particle size improved the extraction of oil from seeds (Del Valle and Uquiche 2002).

Extraction processes showing more than 85% astaxanthin recovery are regarded as the industrial benchmark (Álvarez et al. 2020). By applying different strategies and process conditions, recoveries up to 98.6% were reached from microalgae (Valderrama et al. 2003; Nobre et al. 2006; Di Sanzo et al. 2018; Molino et al. 2018a) (Table 1). An astaxanthin recovery of 90% was also achieved for disrupted *Phaffia rhodozyma* by Lim et al. (2002). All these studies used semi-continuous extraction compared to batch extraction applied in this work. Due to the extraction yields and selectivity determined in this work, it is plausible that applying a multi-stage countercurrent extraction (increasing the driving force for extraction for depleted biomass by bringing it into contact with fresh CO_2_) can also significantly enhance the overall extraction yield.

**Table 1:** Comparison of scCO_2_ extraction processes for astaxanthin extraction. Extraction yields correspond to the reference extraction method used in the respective study

Astaxanthin source	Extraction yield	Extraction conditions	Process mode	Reference
*C. glutamicum* ASTA*	93%	68 °C, 550 bar, 9% (*w*/*w*) ethanol as cosolvent	Batch	This study
*H. pluvialis*	> 97%	60 °C, 300 bar, 9.4% (*w*/*w*) ethanol as cosolvent	Semi-continuous	(Valderrama et al. 2003)
*H. pluvialis*	> 90%	60 °C, 300 bar, 10% (*v*/*v*) ethanol as cosolvent	Semi-continuous	(Nobre et al. 2006)
*H. pluvialis*	99%	50 °C, 550 bar	Semi-continuous	(Di Sanzo et al. 2018)
*H. pluvialis*	92%	65 °C, 550 bar, 12.5% (*v*/*v*) ethanol as cosolvent	Semi-continuous	(Molino et al. 2018a)
*H. pluvialis*	95%	50 °C, 500 bar	Semi-continuous	(Álvarez et al. 2020)
*P. rhodozyma*	90%	40 °C, 500 bar, up to 5% (*v*/*v*) ethanol as cosolvent	Semi-continuous	(Lim et al. 2002)
*Paracoccus sp.* NBRC 101,723	304%	40 °C, 350 bar, 20% (*v*/*w*) ethanol as cosolvent	Semi-continuous	(Chougle et al. 2016)

## Conclusion

In this study, scCO_2_ was employed to extract astaxanthin from the industrially relevant microorganism *C. glutamicum*. Three key findings emerged: (I) the addition of ethanol as a cosolvent was essential to achieve a high yield; (II) temperature influenced the extraction yield more than pressure; and (III) diffusion was identified as the controlling mechanism. Under optimized conditions (9% (*w*/*w*) ethanol, 68 °C, 550 bar, 14 h), a yield of 93.3% was achieved. These results highlight the potential of *C. glutamicum* biomass as a valuable source for natural products like carotenoids, broadening its industrial applications.

## Electronic supplementary material

Below is the link to the electronic supplementary material.

Supplementary Material 1

## Data Availability

The datasets used and/or analysed during the current study are available from the corresponding author on reasonable request.
