# Supplementary material for: Supercritical carbon dioxide extraction of astaxanthin from Corynebacterium glutamicum
Source: Bioresour Bioprocess. 2025 May 26;12(1):46. doi: 10.1186/s40643-025-00882-9 (PMC12106249; doi:10.1186/s40643-025-00882-9)
Supplement: Supplementary file 1 — Supplementary Material 1 [file 40643_2025_882_MOESM1_ESM.docx]

**Supplementary information**

**Supercritical carbon dioxide extraction of astaxanthin from *Corynebacterium glutamicum***

Jan Seeger^1^, Maximilian Zäh^2^, Volker F. Wendisch^1^, Christoph Brandenbusch^2^ and Nadja A. Henke^1,3,*^

^1^ Genetics of Prokaryotes, CeBiTec, Bielefeld University, Bielefeld, Germany

^2^ Laboratory of Thermodynamics, TU Dortmund University, Dortmund, Germany

^3^ Current address: Institute for Process Engineering in Life Sciences, Karlsruhe Institute of Technology (KIT), Karlsruhe, Germany

^*^ Correspondence: [nadja.henke@kit.edu](mailto:nadja.henke@kit.edu)


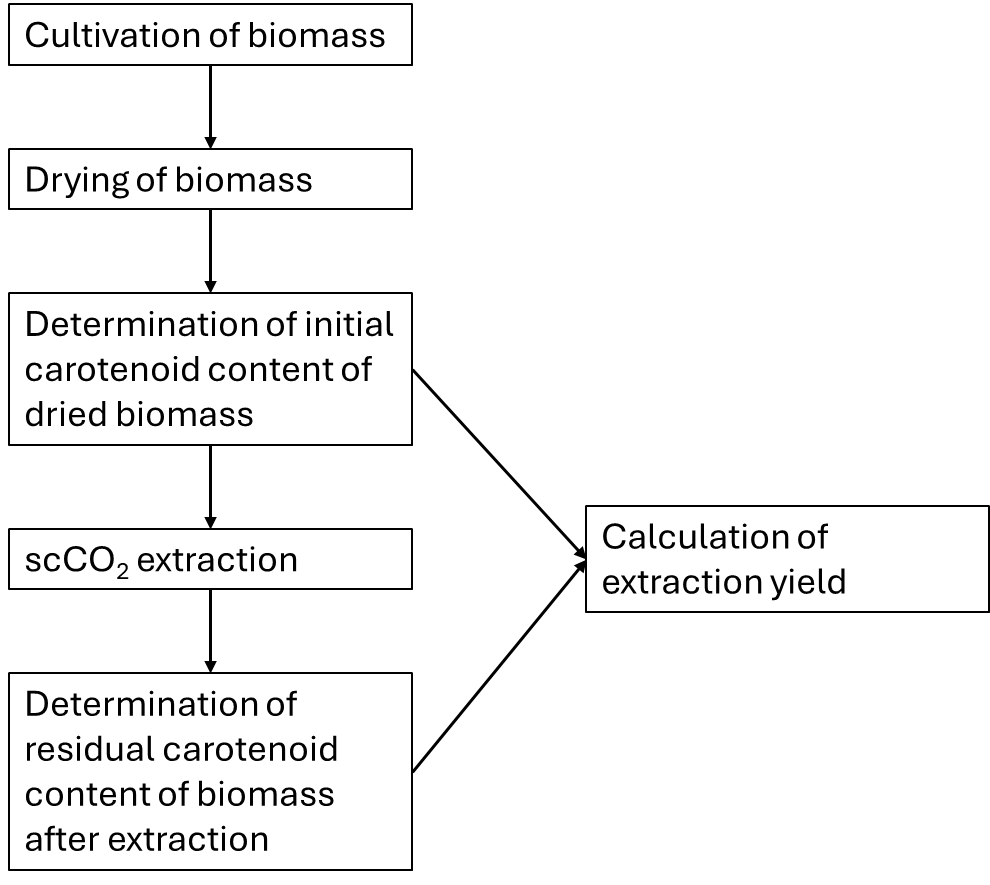


**Figure S1: Experimental workflow to determine extraction yield.** The carotenoid content of the biomass was determined before and after scCO_2_ extraction. Based on that, the extraction yield was calculated.


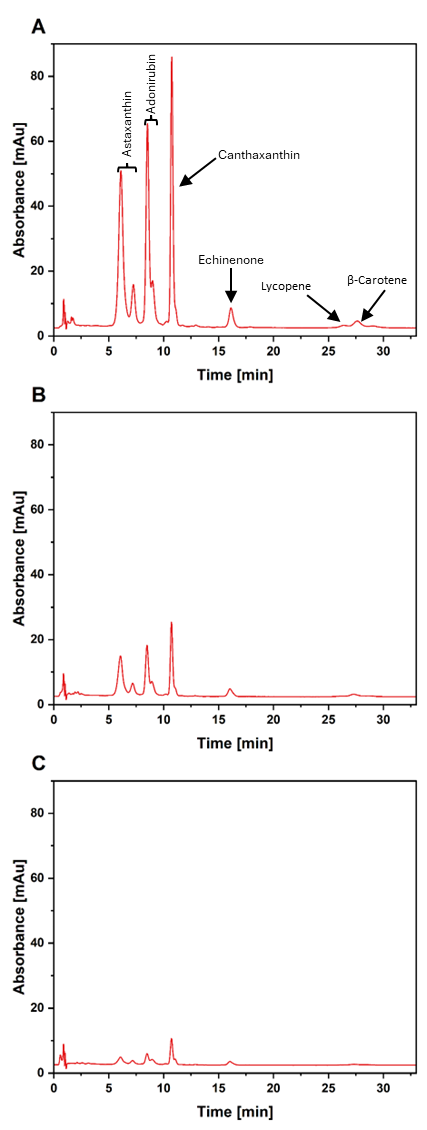


**Figure S2: HPLC chromatograms of carotenoid analysis.** A: Chromatogram of initial carotenoid content. B: Chromatogram after scCO_2_ extraction at 68 °C, 550 bar, 9% (w/w) cosolvent, 0.5 h. C: Chromatogram after scCO_2_ extraction at 68 °C, 550 bar, 9% (w/w) cosolvent, 14 h.

**Table S1: Parameters for scCO_2_ extraction.** All extraction parameters and the corresponding extraction yield are given. All measurements, except the one at 68 °C, 550 bar, 0.5 h, 9% (w/w) cosolvent (n = 3, mean ± sd), were performed as single replicates.

| **Temperature [°C]** | **Pressure [bar]** | **Extraction time [h]** | **Cosolvent  [%** **(*w*/*w*)]** | **Extraction yield [%]** |
| --- | --- | --- | --- | --- |
| 55 | 550 | 0.5 | - | 6.6 |
| 55 | 550 | 0.5 | 4 | 11.5 |
| 55 | 550 | 0.5 | 9 | 42.7 |
| 50 | 550 | 0.5 | 9 | 41.3 |
| 55 | 550 | 0.5 | 9 | 42.7 |
| 60 | 550 | 0.5 | 9 | 57.4 |
| 68 | 550 | 0.5 | 9 | 67.5 ± 3.7 |
| 75 | 550 | 0.5 | 9 | 47.1 |
| 50 | 650 | 0.5 | 9 | 61.5 |
| 55 | 650 | 0.5 | 9 | 47.0 |
| 68 | 650 | 0.5 | 9 | 40.3 |
| 75 | 650 | 0.5 | 9 | 44.6 |
| 68 | 500 | 0.5 | 9 | 44.7 |
| 68 | 450 | 0.5 | 9 | 40.5 |
| 68 | 550 | 14 | - | 56.3 |
| 68 | 550 | 14 | 9 | 93.3 |
| 68 | 550 | 0.5 | - | 16.7 |
